# Design and Realization of Ohmic and Schottky Interfaces for Oxide Electronics

**DOI:** 10.1002/smsc.202100087

**Published:** 2021-11-05

**Authors:** Jie Zhang, Yun-Yi Pai, Jason Lapano, Alessandro R. Mazza, Ho Nyung Lee, Rob G. Moore, Benjamin J. Lawrie, T. Zac Ward, Gyula Eres, Valentino R. Cooper, Matthew Brahlek

**Affiliations:** ^1^ Materials Science and Technology Division Oak Ridge National Laboratory Oak Ridge Tennessee 37831 USA

**Keywords:** epitaxial oxides, oxide electronics, oxide heterostructure, oxide interfaces

## Abstract

Understanding band alignment and charge transfer at complex oxide interfaces is critical to tailoring and utilizing their diverse functionality. Toward this goal, both Ohmic‐ and Schottky‐like charge transfers at oxide/oxide semiconductor/metal interfaces are designed and experimentally validated. A method for predicting band alignment and charge transfer in ABO_3_ perovskites is utilized, where previously established rules for simple semiconductors fail. The prototypical systems chosen are the rare class of oxide metals, SrBO_3_ with B = V–Ta, when interfaced with the multifaceted semiconducting oxide, SrTiO_3_. For B = Nb and Ta, it is confirmed that a large accumulation of charge occurs in SrTiO_3_ due to the higher energy Nb and Ta states relative to Ti. This gives rise to a high mobility metallic interface, which is an ideal epitaxial oxide/oxide Ohmic contact. On the contrary, for B = V, there is no charge transfer into the SrTiO_3_ interface, which serves as a highly conductive epitaxial gate metal. Going beyond these specific cases, this work opens the door to integrating the vast phenomena of ABO_3_ perovskites into a wide range of practical devices.

## Graphical abstract

The diverse functionality of complex oxides can overcome many current technological challenges. Realization, however, necessitates solving the key challenge of tailoring and controlling charge transfer at oxide interfaces. Toward this goal, the authors design and realize both Ohmic‐ and Schottky‐like charge transfers at an important class of oxide/oxide semiconductor/metal interfaces, thus achieving a critical step toward oxide electronics.

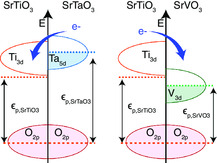

## 1 Introduction

The last half‐century has seen nearly exponential growth in electronic‐based technologies that have permeated and changed the daily lives of nearly all humans. This was driven by the combination of downscaling of the transistor with simultaneous cost reductions and the rapid evolution of a wide array of peripheral devices such as displays, batteries, data transmission, storage, and so on. Currently, however, there are critical obstacles to continued innovation related to fundamental physical size limitations,^[^ ^1^, ^2^ ^]^ as well as societal needs such as energy efficient devices and more robust energy harvesting approaches. Many promising directions to overcome these hurdles are focused on diversifying materials to include systems with functionality beyond traditional metals, insulators, and semiconductors. The ABO_3_ perovskite oxides are viewed as key candidates to compliment or replace current materials in new electronic devices due to the large array of functional properties.^[^ ^3^, ^4^, ^5^ ^]^ These novel aspects are directly tied to the activation of multiple electron degrees of freedom (spin, orbital, and charge), and the much smaller characteristic length scales, such as short screening lengths associated with strong nonlinear dielectric responses at highly charged interfaces, strong localized magnetism, and Coulombic interactions. Furthermore, there are a huge number of elemental combinations that fit onto the perovskite cation sublattices, which exhibit both traditional functionalities and a wide array of additional physical properties; spanning conventional semiconductors, insulators, and metals, to more exotic systems including a wide variety of magnetic materials, ferroelectric, superconductors, and metastable phase‐change materials. These have implications across areas ranging from applications in dielectrics and power electronics, to spintronics, neuromorphic computing, and even superconducting based quantum technologies.

A key difficulty in utilizing the vast functionality of complex oxides is forming structurally and chemically stable heterojunctions, which enable processes such as carrier injection and depletion that form the basis of ubiquitous Ohmic and Schottky barriers. Due to the high offset of the work functions, many noble metals and metal alloys^[^ ^6^ ^]^ form only Schottky barriers in conjunction with prototypical perovskite materials such as SrTiO_3_.^[^ ^7^, ^8^, ^9^ ^]^ Lower work function metals are typically more reactive and adhere better to oxide surfaces, but they often form interfacial oxide layers or intermix with the oxide. For example, key materials that form Ohmic contacts, such as Cr, do so partially because of interfacial diffusion.^[^ ^10^ ^]^ Furthermore, epitaxial integration of metals with perovskites is rare. For example, Pt, Cu (which both form Schottky junctions with SrTiO_3_) and Cr orient the [001] // to the SrTiO_3_ [001], while Ag and Au form textured films.^[^ ^10^, ^11^, ^12^ ^]^ The mismatch between metals and oxides often leads to challenges related to epitaxial relaxation and defect formation, unintended chemical interactions often due to high oxygen diffusion from the perovskite under typical growth temperatures, or nonideal effects related to texturing or high interfacial resistance.^[^ ^10^, ^13^ ^]^ Oxide‐to‐oxide type junctions provide a promising avenue to overcome these issues and give access to a more diverse palette of materials for the design of both high‐quality Ohmic and Schottky barriers.^[^ ^10^ ^]^ This approach also enables new levels of control through epitaxy, which may be applied to realize designer functionalities not accessible in simple metal‐to‐oxide interfaces.^[^ ^14^ ^]^ Identifying a wide range of functionally diverse and robustly compatible oxide candidates that can be epitaxially coupled without disturbing the sharp atomic interface is a critical step toward device integration in complex oxides.^[^ ^15^ ^]^ However, Anderson's and the Schottky–Mott rules, empirically used to predict band alignment and charge transfer based on the energetics of the bulk compounds relative to the vacuum,^[^ ^16^ ^]^ are of limited use for applications in perovskites. This is due to invalid assumptions in general,^[^ ^17^ ^]^ and further complications that arise at oxide/oxide interfaces such as the fundamentally smaller characteristic length scales of charge transfer and microscopic complexities due to bond distortions and atomic rearrangement, and the invalidity of the assumption of a single‐valued work function.^[^ ^9^, ^18^, ^19^ ^]^

Toward this goal, we design and realize two key interface types for electronic devices, the Ohmic‐ and Schottky‐type interfaces at epitaxial oxide/oxide semiconductor/metal junctions. This is achieved by utilizing a new physically accurate band alignment scheme based on the continuity of the oxygen 2*p* states.^[^ ^18^ ^]^ This work centers on the versatile and most widely used perovskite semiconductor SrTiO_3_, with the VB group Sr(V–Ta)O_3_ oxides which are one of the few highly conductive metal perovskites. We predict and experimentally validate that SrTiO_3_/SrVO_3_ forms a Schottky‐type interface, while SrTiO_3_/SrTaO_3_ is Ohmic. Surprisingly, we find that the SrTaO_3_/SrTiO_3_ system gives rise to a conductive state with a residual resistivity ratio (RRR) on the order of 10 000, high mobility, and large magnetoresistance (MR), which makes it an unusually good Ohmic contact material. We demonstrate that the band alignment is dictated by the oxygen continuum boundary condition, which serves as a powerful tool for designing all‐oxide devices around a wide array of novel functional phenomena. We survey an important collection of materials combinations, which will act as a guide to finding and creating new functional devices for next‐generation applications.

## 2 Results

**Figure 1:**
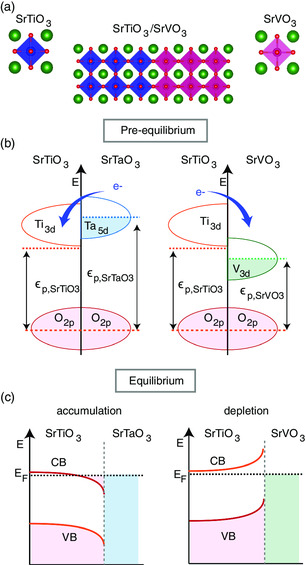
a) Crystal structures of perovskite compounds SrVO_3_ and SrTiO_3_, and the corresponding heterojunction. b) Band alignment in SrBO_3_–SrTiO_3_ heterojunction showing different charge transfer directions of SrVO_3_ versus SrTaO_3_ when interface with SrTiO_3_ based on their bulk energy states and the continuum oxygen state boundary condition as predicted in the study by Zhong and Hansmann.^[^ ^19^ ^]^ c) Schematic of the corresponding band bending that occurs in SrBO_3_–SrTiO_3_ heterojunctions after equilibrium is established, which shows the formation of an Ohmic‐type interface in SrTaO_3_/SrTiO_3_ and a Schottky‐type interface in SrVO_3_/SrTiO_3_.

A recently developed scheme by Zhong and Hansmann for predicting the sign and amplitude of the charge transfer at oxide interfaces is directly associated with the relative energy difference of the oxygen 2*p* (O 2*p*) states in the ABO_3_/AB′O_3_ heterojunctions,^[^ ^18^ ^]^ as shown in **Figure** **1**a for the specific cases of SrTiO_3_ and SrVO_3_. To establish equilibrium, i.e., a constant Fermi energy, charge must be transferred across the interface. As schematically shown in Figure 1b, when the O 2*p* states are aligned the direction of charge transfer is dictated by the difference in Fermi energies, *E*_F_, relative to the aligned O 2*p* states, which is denoted by ε_p_. Applying this theory would suggest, for the specific case here, that as ε_p,SrTaO3_–ε_p,SrTiO3_ > 0, the charge will be transferred from the SrTaO_3_ to the SrTiO_3_, thereby populating the states on the SrTiO_3_ side, as shown in Figure 1c. Alternatively, for the case of ε_p,SrVO3_–ε_p,SrTiO3_ < 0 charge associated with any occupied states on the SrTiO_3_ side will be transferred to the SrVO_3_, thereby depopulating the SrTiO_3_.

The interface among the semiconductor SrTiO_3_ with VB group Sr(V–Ta)O_3_ (semiconductor/metal) provides an ideal test bed to probe the physics of band alignment at oxide interfaces and is of high relevance from an oxide electronics perspective. Within the perovskites, good metals are rare, which highlights the importance of the VB group Sr(V–Ta)O_3_. When going from V to Ta, the work function difference is large enough to cover the energy range needed to form both Schottky (SrVO_3_) and Ohmic (SrTaO_3_) heterojunctions with SrTiO_3_, as schematically shown in Figure 1b,c. In addition, the electronic configuration in the Sr(V–Ta)O_3_ system is relatively simple and does not involve complexities such as magnetism, as is common for the most well‐known perovskite metal, SrRuO_3_. The epitaxial synthesis of SrVO_3_ and SrTaO_3_ is relatively easy due to their crystalline compatibility with SrTiO_3_ because both have simple cubic structures with close to unity tolerance factor^[^ ^20^, ^21^ ^]^ and high chemical stability. Lastly, Sr(V–Ta)O_3_ perovskites represent an important class of functionality where the strong electron–electron correlations open a region of transparency in the visible to ultraviolet portions of the spectrum,^[^ ^22^, ^23^, ^24^ ^]^ which is uncommon for metals in general.

**Figure 2:**
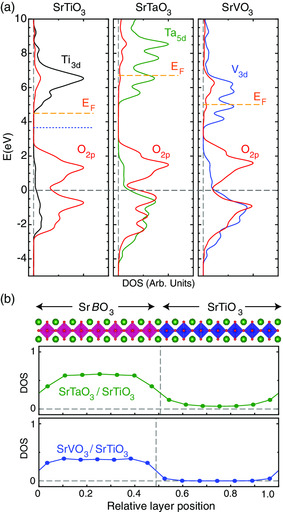
a) Energy‐dependent DOS for bulk SrTiO_3_ (left), SrTaO_3_ (center), and SrVO_3_ (right), where the energy has been shifted to align the O 2*p* states at *E* = 0 eV which is denoted by a dashed gray line. The red curves are the O 2*p* states, and the black/green/blue curves are the transition metal *d* states, as indicated. The orange line indicates the Fermi energy (the dotted blue line for SrTiO_3_ indicates the Fermi energy in the intrinsic limit). b) Spatially resolved layer‐by‐layer # electrons for superlattice systems. Data points indicate B‐site positions within a supercell as indicated by the crystal structure model vertically above. Left of the dashed vertical line is the SrTaO_3_ (top panel)/SrVO_3_ (bottom panel) side of the interface, and the right side is the SrTiO_3_. The nonzero number of electrons on the SrTiO_3_ indicates the presents of a 2DEG due to charge transfer into the SrTiO_3_ for the case of SrTaO_3_, but not for SrVO_3_.

To go beyond this schematic argument, we performed first‐principles calculations for the SrTaO_3_/SrTiO_3_ and SrVO_3_/SrTiO_3_ systems with the results being shown in **Figure** **2**. (see Supporting Information for calculation details which include Ref. [25, 26]). The energy‐dependent density of states (DOS) was calculated for all three systems in a bulk‐like configuration and the energy was shifted to align the weighted‐band center of the O 2*p* states, as shown in Figure 2a. Here, we show the Fermi energy for SrTiO_3_ in the intrinsic limit (i.e., the Fermi level is within the gap in the limit of no charge doping), as well as near the bottom of the conduction band, which is the experimentally relevant point because bulk SrTiO_3_ ubiquitously exhibits electron‐doped oxygen vacancies. (This rigid‐band shift for bulk SrTiO_3_ is valid in the dilute limit; however, at interfaces there may be a larger density of oxygen vacancies that may locally distort the O 2*p* states, which could modify the charge transfer profile). Based on the relative position of the Fermi energies (i.e., the relative values of ε_p_), it can be surmised that SrTaO_3_ will transfer charge to SrTiO_3_, thus creating an accumulation layer (identical results are found for SrNbO_3_; see Supporting Information). For SrVO_3_, we find that the values of ε_p_ are relatively close to that of SrTiO_3_, which necessitates a more realistic set of calculations to determine the charge transfer direction. Therefore, we performed heterostructure calculations where seven unit cells of both SrTaO_3_ and SrVO_3_ were interfaced with seven unit cells of SrTiO_3_. The results for these calculations are shown in Figure 2b where the number of free electrons (related to the layer‐resolved density of states) was determined for the individual atomic layers across the entirety of the heterostructure. For both systems, the number of electrons on the left side is nonzero over the entirety of the thickness, which is consistent with the metallic nature of these materials. However, for the SrTiO_3_ side, there is a significant difference. For SrTaO_3_/SrTiO_3_, the number of electrons on the SrTiO_3_ side is nonzero, which indicates that there is an emergent 2D electron gas (2DEG) due to charge transfer.^[^ ^27^, ^28^, ^29^, ^30^ ^]^ However, for the SrVO_3_/SrTiO_3_, the number of electrons on the SrTiO_3_ side is zero, indicating there is no charge transfer into the SrTiO_3_ and no 2DEG exists. The important feature here is that unlike the Sr(Ta/Nb)O_3_/SrTiO_3_ superlattices, in the case of SrVO_3_/SrTiO_3_ the occupied V *d* states are lower in energy relative to the center of the oxygen band than the Ti *d* states. This would suggest that the electrons should remain in the V *d* bands rather than being transferred to the Ti *d* states. Therefore, first‐principles calculations predict that SrVO_3_–SrTaO_3_ on SrTiO_3_ span Ohmic‐like to Schottky‐like charge transfer and are experimentally accessible.

Our first‐principles calculations have shown that charge transfers into SrTiO_3_ from Sr(Ta/Nb)O_3_ due to the relative band alignment, which gives rise to an interfacial 2DEG. This provides a simple route to test the band alignment of SrTiO_3_ relative to SrVO_3_ and SrTaO_3_ using simple low‐temperature transport. As SrVO_3_ does not transfer charge into SrTiO_3_, no 2DEG should form and conduction should be solely through the SrVO_3_ film. On the contrary, in the case of SrTaO_3_ a 2DEG should form, which will conduct in parallel with the SrTaO_3_. 2DEGs in SrTiO_3_ are characterized by extremely high mobilities, in excess of 100 000 cm^2^ V^−1^ s^−1^, carrier densities in the range of 10^13^–10^15^ cm^−2^/10^19^–10^20^ cm^−3^,^[^ ^31^ ^]^ and an accumulation length that is typically of the scale of 5 nm dictated by the high dielectric constant of SrTiO_3_. This combination of high mobility and high interfacial carrier density gives rise to very large conductivity, which may be comparable with the conductivity of the perovskite metal (SrTaO_3_). This is due to the fact that, despite the carrier density in SrTaO_3_ being of order 10^22^ cm^−3^,^[^ ^32^ ^]^ mobilities are typically quite low for pulsed laser deposition (PLD)‐grown perovskite metal films due to a large effective mass combined with short scattering times due to typical dominance of strong surface scattering,^[^ ^33^ ^]^ uncontrolled nonstoichiometry,^[^ ^34^ ^]^ or both.

**Figure 3:**
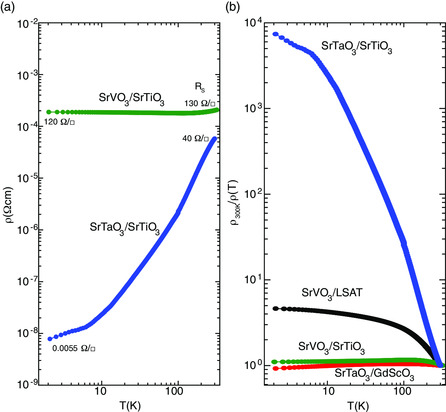
a) Temperature dependence of longitudinal resistivities for SrVO_3_/SrTiO_3_ (16 nm) and SrTaO_3_/SrTiO_3_ (14 nm), and indicated are the sheet resistance, *R*_S_, values at room temperature and 2 K (see Figure S4, Supporting Information, for a plot of *R*_S_ vs *T*). b) *ρ*_300 K_/*ρ*(*T*) for SrVO_3_/SrTiO_3_ and SrTaO_3_/SrTiO_3_ shown in (a) as well as films grown on highly insulating SrVO_3_/LSAT and SrTaO_3_/GdScO_3_ demonstrating only SrTaO_3_–SrTiO_3_ exhibits a high residual resistivity ratio of order of 10 000 due to the Ohmic‐type interface.

**Figure** **3** shows a compilation of temperature‐dependent transport measurements for SrVO_3_ and SrTaO_3_ films grown by PLD on SrTiO_3_ as well as highly insulating LSAT and GdScO_3_. Here, the films were grown using a KrF excimer laser with 5 Hz repetition rate, a fluence of 0.5 J cm^−2^ at a temperature of 650 °C in oxygen partial pressure of 4 × 10^−6^ Torr (see Supporting Information section 1 for details regarding the synthesis). The structure was characterize using X‐ray diffraction and found to be epitaxially strained for all films. The transport measurements were performed in the standard van der Pauw geometry with Ohmic indium contacts. Figure 3a shows the temperature dependence of the resistivity for both SrVO_3_ and SrTaO_3_ on SrTiO_3_. This is obtained by multiplying the sheet resistance, *R*_S_, multiplied by the film thickness (*R*_S_ is indicated in Figure 3a and Figure S4, Supporting Information). Both materials exhibit resistivities that decrease with decreasing temperature, which indicates that both systems are metals. Although qualitatively similar, the two systems are quantitatively different in that the low‐temperature resistivity of the SrTaO_3_ system is orders‐of‐magnitude smaller than the SrVO_3_ system. SrVO_3_ has a low‐temperature resistivity of 2 × 10^−4^ Ω cm, whereas the SrTaO_3_/SrTiO_3_ system exhibits a nominal low‐temperature resistivity four orders of magnitude smaller at around 8 × 10^−9^ Ω cm. In contrast, the room‐temperature resistivities are similar at around 1 × 10^−4^ Ω cm. It is customary to use the residual resistivity ratio, RRR = ρ(T=300K)/ρ(T=2K), as a metric of the concentration of defects within a materials class, which can be seen in Figure 3b where ρ(T=300K)/ρ(T) is plotted. For example, hybrid molecular beam epitaxy‐grown SrVO_3_ films with a high level of control over the stoichiometry can reach a RRR over 200,^[^ ^20^, ^35^, ^36^ ^]^ which is likely limited only by surface scattering. In contrast, films grown by PLD typically possess lower RRRs due to higher defect concentrations incorporated during synthesis. Our PLD‐grown SrVO_3_ exhibits RRR on the order of 1 when grown on SrTiO_3_ and ≈4 when grown on highly insulating LSAT (the slightly larger RRR may be due to better lattice matching on LSAT than on SrTiO_3_). In contrast, SrTaO_3_ grown under identical conditions exhibits RRRs around 10 000, as shown in Figure 3b. It is tempting to conclude that the SrTaO_3_ heterostructure is of much higher quality than the SrVO_3_. However, growing identical SrTaO_3_ films on highly insulating GdScO_3_ reveals a RRR similar to that of SrVO_3_. This indicates that the single‐slab‐interpretation is not valid. Instead, one must consider the entire interfacial SrVO_3_/SrTiO_3_ and SrTaO_3_/SrTiO_3_ systems.

To see that the interface with SrTiO_3_ must be playing a role, we consider the resistivities of SrTaO_3_ grown on highly insulating GdScO_3_ relative to that of bulk SrTiO_3_ versus doping levels.^[^ ^37^, ^39^ ^]^ The resistivity of SrTaO_3_ on GdScO_3_ is found to be nearly temperature independent at around 1 × 10^−4^ Ω cm, several orders larger than on SrTiO_3_. The low‐temperature resistivity in bulk SrTiO_3_ (doped with either Nb or oxygen vacancies) is typically found to have a minimum at 10^−3^–10^−4^ Ω cm at a doping greater than 10^16^–10^17^ cm^−3^. Carrier densities due to oxygen vacancies of this range can easily be achieved by annealing SrTiO_3_ in vacuum at temperatures similar to those used to grow the films in this work, 650 °C. Therefore, assuming that the band alignment theory is correct implies that the transport is likely through the SrTaO_3_ and the SrTaO_3_/SrTiO_3_ interface, and possibly the bulk SrTiO_3_. If this is so, the measured resistivity would be given by the summation of the parallel conductance channels, *G*_Total_ *= G*_SrTiO3_ + *G*_2DEG_ *+ G*_SrTaO3_, where each conductance channel is given by *eμn*_2D_, where *e* is the electron charge, *μ* is the mobility, and *n*_2D_ is the areal carrier density of each channel (*n*_3D_ × thickness), respectively. Experimentally, measuring the net resistivity is given by *G*_Total_^−1^ multiplied by the thickness of SrTaO_3_. Given typical mobilities and carrier densities for bulk SrTiO_3_ and interfacial 2DEGs, this yields effective resistivities on the order of 10^−8^–10^−9^ Ω cm at 2 K and 10^−4^ at room temperature, which identically matches the experimental data shown in Figure 3. As the SrVO_3_ and SrTaO_3_ systems were grown under identical conditions (temperature and oxygen partial pressures), they should have identical SrTiO_3_ doping levels. Thus, we can conclude that the SrTaO_3_/SrTiO_3_ system forms an Ohmic junction, whereas the SrVO_3_/SrTiO_3_ system forms a Schottky junction.

**Figure 4:**
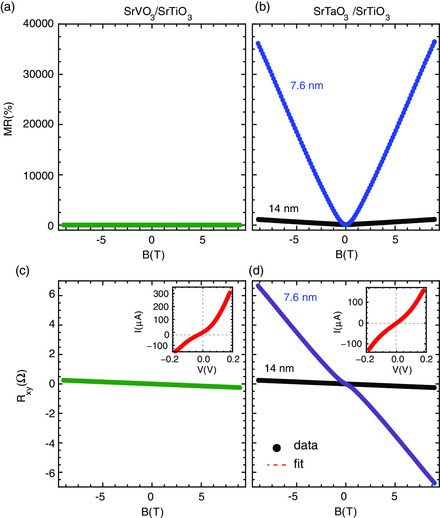
Transport data of selected SrVO_3_/SrTiO_3_ (left panels) and SrTaO_3_/SrTiO_3_ (right panels). a,b) MR showing MR ≈0.1% in SrVO_3_/SrTiO_3_ (a) but nearly 40 000% in SrTaO_3_/SrTiO_3_ (b). c,d) Hall effect, where SrVO_3_ shows a linear dependence (c), whereas SrTaO_3_/SrTiO_3_ shows nonlinear behavior (d). See Supporting Information for multicarrier fitting and detailed analysis and plots of *dR*_*xy*_/*dB* which highlight the nonlinear Hall effect. Insets in (c,d) are current voltage (*I*–*V*) curves for similar samples grown on Nb‐doped SrTiO_3_ with doping level 0.5%.

Magnetotransport measurements give additional insights into the origin and character of the strikingly different behaviors of the SrVO_3_/SrTiO_3_ and SrTaO_3_/SrTiO_3_ systems and point to the formation of an interfacial 2DEG. The magnetoresistance (MR = [*ρ*(*B*)–*ρ*(*B* = 0 T)]/*ρ*(*B* = 0 T) × 100%) is shown in **Figure** **4**a,b and the Hall resistance is shown in Figure 4c,d for both SrVO_3_/SrTiO_3_ and SrTaO_3_/SrTiO_3_, respectively. We first discuss the MR and then the Hall effect. Due to the orbital motion, a semiclassical picture of the MR of a metal (strictly in a multiband case) is a function of the mobility, MR ≈ (*μB*)^2^,^[^ ^39^ ^]^ which is in the low‐field limit (*μB* < 1). However, for higher magnetic fields the MR should saturate for a semiclassical model or continue to grow unbounded if more complex physics is at play.^[^ ^39^ ^]^ Here, we see that the SrVO_3_ system exhibits only low‐field orbital MR (MR ≈ (*μB*)^2^), which is maximum at 0.10% at 9 T and 2 K. This reflects the low film mobility and the absence of any substrate effects. In contrast, the behavior of SrTaO_3_ is more complex. In the low‐field limit, MR ≈ (*μB*)^2^ is dominated by the orbital motion at all temperatures. For low temperatures, the MR rises to 1000% at around 1–2 T, which is consistent with mobilities on the order of 5000–10 000 cm^2^ V^−1^ s^−1^. In the high‐field limit, however, the MR grows roughly linearly to the maximum field used, 9 T, to a value of nearly 40 000%. To fully explain this behavior requires more complex transport models and will be a question of future interest. Nevertheless, this data points to the combination of a high‐mobility state in conjunction with a low mobility state, which can be further elucidated in the context of the Hall effect data.

For a semiclassical single‐band metal, the Hall slope is a direct measure of the density of charge carriers. In the limit of multiple bands or systems with complex Fermi surface topologies, the interpretation is more intricate but can provide a semiquantitative characterization of the metallic state. SrVO_3_, SrTaO_3_, bulk SrTiO_3_, and 2DEG systems can exhibit multiband effects only when the mobility is sufficiently high. In the SrVO_3_ system, *R*_*xy*_ is linear and temperature independent, with a slope that yields a carrier density that is close to the single‐band carrier density (1 electron per unit cell ≈10^22^ cm^−3^). This reflects the overall low mobility and the fact that the transport is solely through the SrVO_3_ film. In contrast, *R*_*xy*_ for SrTaO_3_ is nonlinear and strongly temperature dependent. This is indicative of multiple carriers with disparate carrier densities and mobilities. The qualitative field dependence of *R*_*xy*_ (low‐field slope smaller than the high‐field slope) indicates one of the dominant carriers to be hole‐like, which, despite no physical hole‐like pockets, can be attributed to portions of the Fermi surface with negative curvature.^[^ ^40^, ^41^ ^]^ As discussed in the Supporting Information, the field and temperature dependence of the Hall effect can be modeled as a three‐carrier system composed of low mobility SrTaO_3_, the high mobility SrTiO_3_, and an interfacial 2DEG. The hole‐like band is chosen to be the 2DEG because bulk SrTiO_3_ in the carrier density range of 10^16^–10^17 ^cm^−3^ is only known to exhibit electron‐like Hall slopes.^[^ ^42^ ^]^ However, high‐mobility 2DEG systems on SrTiO_3_ commonly exhibit both electron‐ and hole‐like multicarrier Hall slopes, which likely point to a complex interfacial Fermi surface which is electronically active and plays a non‐negligible effect in the overall conductance. The results of the fitting yield an SrTiO_3_ carrier density in the range of 10^16^ cm^−3^, which exactly matches the expected carrier range for the growth temperatures used.^[^ ^37^ ^]^ Furthermore, the carrier density for the 2DEG is in the range of 10^13^ cm^−2^, which is again consistent with both the expected charge transfer at an oxide interface and with known 2DEGs on SrTiO_3_. The mobilities are of the range of several thousand which match previous measurements.

The SrTaO_3_/SrTiO_3_ system exhibits a rich array of transport phenomena that indicate the conduction is through both the SrTaO_3_, an interfacial 2DEG, and the bulk SrTiO_3_ as would be expected in an Ohmic‐type contact. The SrVO_3_/SrTiO_3_ system presents more straightforward transport solely through the SrVO_3_ layer as expected for a Schottky contact. Ohmic point contacts with a diameter of <500 μm were applied to the film surface and back of the substrate to conduct current–voltage (*I–V*) measurements across the interface for both systems grown on Nb‐doped SrTiO_3_ substrates with doping at 0.5%. The results are shown in the insets of Figure 4c,d. Here, the *I*–*V* curves for SrVO_3_/SrTiO_3_ show an asymmetric character with a much steeper slope on the positive bias side. This is a classic indicator of Schottky‐like charge transfer. In contrast, the SrTaO_3_ shows a symmetric and nearly linear *I*–*V* character, which is indicative of an Ohmic‐like interface. This is in agreement with transport results as well as the theory for constructing band alignments in oxide systems.

## 3 Discussion

These findings provide experimental evidence that support the theory for band alignment of multicomponent oxides, which predicts that the SrTaO_3_/SrTiO_3_ system forms an Ohmic junction, whereas SrVO_3_/SrTiO_3_ forms a Schottky junction. This observation is critical to expanding into all oxide electronics which can make use of the great diversity of phenomena known in the multicomponent oxides. Specifically, this provides a route to design energy‐level alignments that can be used to dictate charge transfer and local electric fields. This ability establishes a clear path to new levels of functional tunability in oxide electronics. Key examples for future work center on materials with traditional functionality such as semiconductors and more exotic materials. For semiconductor devices, there is a particular importance of SrTiO_3_,^[^ ^4^ ^]^ which has a large and highly tunable dielectric constant^[^ ^43^ ^]^ and lattice matching to Si and other semiconductors,^[^ ^3^ ^]^ but also other dielectrics such as (Sr,Ba)TiO_3_^[^ ^44^ ^]^ and Sr_*n*+1_Ti_*n*_O_4_,^[^ ^45^ ^]^ as well as SrZrO_3_ and SrHfO_3_ which also have highly tunable dielectric responses. Here, for SrTiO_3_, Sr(V–Ta)O_3_ are likely good choices to engineer desired interfacial properties, where other perovskite metals (SrRuO_3_ and SrIrO_3_) are predicted to only form Schottky junctions. Interestingly, theory predicts that for SrZrO_3_ only SrTaO_3_ is Ohmic, while for SrHfO_3_, Sr(V–Ta)O_3_ are all Schottky‐like. Extending this scheme outside of transition metals to include the Sn‐based perovskites such as BaSnO_3_ is of significant interest because the extended *p*‐bands of Sn give rise to relatively wide bandgaps and large room‐temperature mobility, which is critical for room‐temperature applications, especially in the realm of power electronics.^[^ ^46^, ^47^ ^]^ Creating well‐controlled high‐quality interfaces and epitaxial contacts to the stannates is an open question for future work.

Beyond these traditional functionalities a host of other useful properties are known, for which creating and controlling the interfacial charge transfer is crucial. Novel magnetic states abound across the transition metals, which can be highly tunable via interfacial charge transfer.^[^ ^18^ ^]^ A key example being SrRuO_3_ (*ε*_p_ = −3.40 eV), which is a ferromagnetic metal and would be an ideal candidate for spin‐injection into a high mobility 2DEG in SrTiO_3_, SrZrO_3_, and SrHfO_3_ (*ε*_p_ = −4.00, −6.23, −7.43 eV, respectively). However, predictions show that these interfaces are Schottky‐like. Therefore, additional work to modify the interface is required to realize such a device. While much work has been done to engineer chiral magnetic interactions at oxide interfaces, it is predominately focused on hybridizing the strong spin–orbit coupling in the metal SrIrO_3_ with magnetic insulators such as SrMnO_3_ and LaMnO_3_, where the electronic states at the interfaces are required to break inversion symmetry.^[^ ^48^ ^]^ Magnetic interactions may be easily controlled by engineering charge transfer across the interface to drive symmetry breaking in SrIrO_3_. Lastly, many perovskites are ideal platforms for energy‐efficient neuromorphic computing that emerge from a strong interplay of metal–insulator transitions and externally controlled oxygen migration, as demonstrated in the rare‐earth nickelates.^[^ ^49^ ^]^ Such processes rely on overcoming built in energy barriers at interfaces and therefore represent a wide array of possible tunability through designing interfacial charge transfer that may be enabled using informed oxide junction selection.

## 4 Conclusion

To conclude, a necessary step toward oxide electronics is the ability to design, tailor, and create charge transfer at epitaxial oxide/oxide interfaces. Toward this goal, we show that Ohmic‐ and Schottky‐like interfaces can be designed and realized via a novel band alignment scheme developed for the multifunctional transition metal oxides. This is demonstrated for the important class of perovskite metals (Sr(V–Ta)O_3_) interfaced with the multifaceted semiconducting oxide, SrTiO_3_. Here, the charge associated with occupied states in SrTiO_3_ is transferred to the SrVO_3_, while SrTaO_3_ creates an accumulation of charge within the SrTiO_3_ interfacial layers. As only a handful of perovskites are good metals, the SrVO_3_–SrTaO_3_ series are a critical class for providing top or bottom gates, as well as highly conductive contact materials. Also, due to the strong electron–electron correlations these materials are highly transparent which make them relatively straightforward building blocks for oxide electronics. Going forward we have discussed key examples and open questions where understanding band alignment and charge transfer at complex oxide interfaces is critical to designing and utilizing their diverse functionalities. Mastering these is key toward realizing and overcoming several challenges facing the continued evolution of electronics.

## Conflict of Interest

The authors declare no conflict of interest.

## Data Availability Statement

The data that support the findings of this study are available from the corresponding author upon reasonable request.

## Supporting information

Supplementary Material

## References

[1] J. S. D. G. Mannhart , Science 2010, 327, 1607. 20339065 10.1126/science.1181862

[2] H. Ilatikhameneh , T. Ameen , B. Novakovic , Y. Tan , G. Klimeck , R. Rahman , Sci. Rep. 2016, 6, 1. 27538849 10.1038/srep31501 PMC4990915

[3] D. P. Kumah , J. H. Ngai , L. Kornblum , Adv. Funct. Mater. 2020, 30, 1901597.

[4] L. Kornblum , Adv. Mater. Interfaces 2019, 6, 1900480.

[5] M. Brahlek , L. Zhang , J. Lapano , H.-T. Zhang , R. Engel-Herbert , N. Shukla , S. Datta , H. Paik , D. G. Schlom , MRS Commun. 2017, 7, 27.

[6] D. E. Eastman , Phys. Rev. B 1970, 2, 1.

[7] M. Minohara , R. Yasuhara , H. Kumigashira , M. Oshima , Phys. Rev. B 2010, 81, 235322.

[8] W. Maus-Friedrichs , M. Frerichs , A. Gunhold , S. Krischok , V. Kempter , G. Bihlmayer , Surf. Sci. 2002, 515, 499.

[9] R. Jacobs , J. Booske , D. Morgan , Adv. Funct. Mater. 2016, 26, 5471.

[10] S. A. Chambers , M. Gu , P. V. Sushko , H. Yang , C. Wang , N. D. Browning , Adv. Mater. 2013, 25, 4001. 23649872 10.1002/adma.201301030

[11] J. Son , J. Cagnon , S. Stemmer , J. Appl. Phys. 2009, 106, 43525.

[12] A. J. Francis , P. A. Salvador , J. Mater. Res. 2007, 22, 89.

[13] Q. Fu , T. Wagner , Surf. Sci. Rep. 2007, 62, 431.

[14] J. M. Rondinelli , S. J. May , J. W. Freeland , MRS Bull. 2012, 37, 261.

[15] N. Nakagawa , H. Y. Hwang , D. A. Muller , Nat. Mater. 2006, 5, 204.

[16] J. H. Davies , The Physics of Low Dimensional Semiconductors, Cambridge University Press, New York 1998.

[17] D. Cahen , A. Kahn , Adv. Mater. 2003, 15, 271.

[18] Z. Zhong , P. Hansmann , Phys. Rev. X 2017, 7, 011023.

[19] Z. Zhong , P. Hansmann , Phys. Rev. B 2016, 93, 235116.

[20] M. Brahlek , L. Zhang , C. Eaton , H. T. Zhang , R. Engel-Herbert , Appl. Phys. Lett. 2015, 107, 143108.

[21] S. A. Khandy , D. C. Gupta , RSC Adv. 2016, 6, 48009.

[22] L. Zhang , Y. Zhou , L. Guo , W. Zhao , A. Barnes , H.-T. Zhang , C. Eaton , Y. Zheng , M. Brahlek , H. F. Haneef , N. J. Podraza , M. H. W. Chan , V. Gopalan , K. M. Rabe , R. Engel-Herbert , Nat. Mater. 2016, 15, 204. 26657329 10.1038/nmat4493

[23] Y. Park , J. Roth , D. Oka , Y. Hirose , T. Hasegawa , A. Paul , A. Pogrebnyakov , V. Gopalan , T. Birol , R. Engel-Herbert , Commun. Phys. 2020, 3, 1.

[24] J. Roth , A. Paul , N. Goldner , A. Pogrebnyakov , K. Agueda , T. Birol , N. Alem , R. Engel-Herbert , ACS Appl. Mater. Interfaces 2020, 12, 30520. 32515187 10.1021/acsami.0c04854

[25] V. R. Cooper , S. S. A. Seo , S. Lee , J. S. Kim , W. S. Choi , S. Okamoto , H. N. Lee , Sci. Rep. 2014, 4, 1. 10.1038/srep06021 PMC4127498 25109668

[26] P. Giannozzi , S. Baroni , N. Bonini , M. Calandra , R. Car , C. Cavazzoni , D. Ceresoli , G. L. Chiarotti , M. Cococcioni , I. Dabo , A. D. Corso , S. de Gironcoli , S. Fabris , G. Fratesi , R. Gebauer , U. Gerstmann , C. Gougoussis , A. Kokalj , M. Lazzeri , L. Martin-Samos , N. Marzari , F. Mauri , R. Mazzarello , S. Paolini , A. Pasquarello , L. Paulatto , C. Sbraccia , S. Scandolo , G. Sclauzero , A. P. Seitsonen , A. Smogunov , P. Umari , R. M. Wentzcovitch , J. Phys. Condens. Matter 2009, 21, 395502. 21832390 10.1088/0953-8984/21/39/395502

[27] A. Ohtomo , H. Y. Hwang , Nature 2004, 427, 423. 14749825 10.1038/nature02308

[28] S. Okamoto , A. J. Millis , Nature 2004, 428, 630. 15071589 10.1038/nature02450

[29] A. F. Santander-Syro , O. Copie , T. Kondo , F. Fortuna , S. Pailhès , R. Weht , X. G. Qiu , F. Bertran , A. Nicolaou , A. Taleb-Ibrahimi , P. Le Fèvre , G. Herranz , M. Bibes , N. Reyren , Y. Apertet , P. Lecoeur , A. Barthélémy , M. J. Rozenberg , Nature 2011, 469, 189. 21228872 10.1038/nature09720

[30] V. R. Cooper , Phys. Rev. B 2012, 85, 235109.

[31] Y. Z. Chen , N. Bovet , F. Trier , D. V. Christensen , F. M. Qu , N. H. Andersen , T. Kasama , W. Zhang , R. Giraud , J. Dufouleur , T. S. Jespersen , J. R. Sun , A. Smith , J. Nygård , L. Lu , B. Büchner , B. G. Shen , S. Linderoth , N. Pryds , Nat. Commun. 4, 2013. 10.1038/ncomms2394 23340411

[32] D. Oka , Y. Hirose , H. Kamisaka , T. Fukumura , K. Sasa , S. Ishii , H. Matsuzaki , Y. Sato , Y. Ikuhara , T. Hasegawa , Sci. Rep. 2014, 4, 1.

[33] G. Koster , D. H. A. Blank , J. Supercond. Nov. Magn. 2020, 33, 205.

[34] D. Oka , Y. Hirose , S. Nakao , T. Fukumura , T. Hasegawa , Phys. Rev. B 2015, 92, 1.

[35] J. A. Moyer , C. Eaton , R. Engel-Herbert , Adv. Mater. 2013, 25, 3578. 23703901 10.1002/adma.201300900

[36] M. Brahlek , A. Sen Gupta , J. Lapano , J. Roth , H. Zhang , L. Zhang , R. Haislmaier , R. Engel-Herbert , Adv. Funct. Mater. 2018, 28, 1702772.

[37] A. Spinelli , M. A. Torija , C. Liu , C. Jan , C. Leighton , Phys. Rev. B 2010, 81, 155110.

[38] X. Lin , B. Fauqué , K. Behnia , Science 2015, 349, 945. 26315430 10.1126/science.aaa8655

[39] A. B. Pippard , Magnetoresistance in Metals, Cambridge University Press, Cambridge 1989.

[40] C. M. Hurd , The Hall Effect in Metals and Alloys, Plenum Press, New York 1972.

[41] N. P. Ong , Phys. Rev. B 1991, 43, 193. 10.1103/physrevb.43.193 9996203

[42] A. Bhattacharya , B. Skinner , G. Khalsa , A. V. Suslov , Nat. Commun. 2016, 7, 1. 10.1038/ncomms12974 PMC5056415 27680386

[43] K. A. Müller , H. Burkard , Phys. Rev. B 1979, 19, 3593.

[44] A. Feteira , D. C. Sinclair , I. M. Reaney , Y. Somiya , M. T. Lanagan , J. Am. Ceram. Soc. 2004, 87, 1082.

[45] C. H. Lee , N. D. Orloff , T. Birol , Y. Zhu , V. Goian , E. Rocas , R. Haislmaier , E. Vlahos , J. A. Mundy , L. F. Kourkoutis , Y. Nie , M. D. Biegalski , J. Zhang , M. Bernhagen , N. A. Benedek , Y. Kim , J. D. Brock , R. Uecker , X. X. Xi , V. Gopalan , D. Nuzhnyy , S. Kamba , D. A. Muller , I. Takeuchi , J. C. Booth , C. J. Fennie , D. G. Schlom , Nature 2013, 502, 532. 24132232 10.1038/nature12582

[46] A. Prakash , B. Jalan , Adv. Mater. Interfaces 2019, 6, 1900479.

[47] W. Nunn , T. K. Truttmann , B. Jalan , J. Mater. Res 2021, 2021, 1.

[48] E. Skoropata , J. Nichols , J. M. Ok , R. V. Chopdeka , E. S. Choi , A. Rastogi , C. Sohn , X. Gao , S. Yoon , T. Farmer , Sci. Adv. 2020, 6, eaaz3902. 32923583 10.1126/sciadv.aaz3902 PMC7455502

[49] J. Shi , S. D. Ha , Y. Zhou , F. Schoofs , S. Ramanathan , Nat. Commun. 2013, 4, 1. 10.1038/ncomms3676 24177330

