# Supplementary material for: Design and Realization of Ohmic and Schottky Interfaces for Oxide Electronics
Source: Small Sci. 2021 Nov 5;2(2):2100087. doi: 10.1002/smsc.202100087 (PMC11936060; doi:10.1002/smsc.202100087)
Supplement: Supplementary file 1 — Supplementary Material [file SMSC-2-2100087-s001.pdf]

Supporting Information
for Small Science., DOI ******

Design and realization of Ohmic and Schottky interfaces for oxide electronics

Supporting Information

Design and realization of Ohmic and Schottky interfaces for oxide electronics

Jie Zhang[1], *Yun-Yi Pai*[1], *Jason Lapano*[1], *Alessandro R. Mazza*[1], *Ho Nyung Lee*[1], *Rob Moore*[1], *Benjamin J. Lawrie*[1], *T. Zac Ward*[1], *Gyula Eres*[1], *Valentino R. Cooper*[1#], *Matthew Brahlek*[1]*

[1]Oak Ridge National Laboratory, Oak Ridge, Tennessee, U.S.A.

Email: *brahlekm@ornl.gov, [#]coopervr@ornl.gov

Film growth

Epitaxial $SrVO_3$ and $SrTaO_3$ thin films were grown on $SrTiO_3$ (Crystec Corporation) using pulsed laser deposition (PLD). Prior to growth the substrates were chemically etched with buffered hydrofluoric acid before annealing in air at 1000 ºC for 2 hours, to achieve nominal TiO_2 termination and atomically flat surfaces. A KrF excimer laser (λ = 248 nm) was used to ablate stoichiometric targets at a repetition rate of 5 Hz and fluence of 0.5 $\mathrm{J/cm^2}$, an oxygen partial pressure of 4×10^{-6} Torr, and a growth temperature of 650 ºC. Crystallinity was examined with X-ray diffraction (XRD) using a four-circle diffractometer (Panalytical Corporation) with Cu-$k_{\alpha 1}$ radiation as shown in Figure S1. Reciprocal space maps demonstrate that the films were fully strained. The out-of-plane lattice constants were extracted from the 002 peak position (c = 3.80 Å for $SrVO_3$ and c = 4.01 Å for $SrTaO_3$). Film thickness was confirmed by X-ray reflectivity (XRR). Transport measurements were performed in a physical property measurement system (PPMS, Quantum Design) using van der Pauw sample configuration by soldering indium contacts.

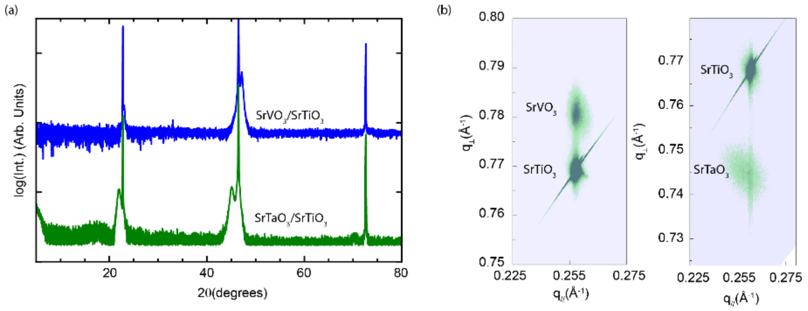

Figure S1. X-ray diffraction analysis of $SrVO_3/SrTiO_3$ and $SrTaO_3/SrTiO_3$ showing 2θ-θ scans about the 00L reflections (a), as well as reciprocal space maps about the 103 set of reflections (b).

First Principles Calculations

All density functional theory calculations were performed using the QUANTUM ESPRESSO simulation code (v 6.4.1) [1] with the local density approximation (LDA) for exchange and correlation and ultrasoft pseudopotentials. The Sr 4s4p5s, Ti 3p3d4s, V 4s3d, Nb 4p4d5s, Ta 5d6s and O 2s2p electrons were treated as valence electrons. A 500 eV cutoff and an 8×8×8 or 8×8×1 Monkhorst-Pack *k*-point mesh was employed for the bulk or superlattice calculations, respectively. A Hubbard U of 5.0 eV for Ti, V, Nb, and Ta d-states was found to be appropriate for all calculations. Similar U values were found to give a reasonable description of the electronic and structural properties of related 2DEG model systems. [2–4] The computed bulk $SrVO_3$, $SrNbO_3$, $SrTaO_3$, and $SrTiO_3$ cubic lattice constants of 3.757, 3.987, 3.971, and 3.879 Å, respectively, are in typical LDA agreement with the experimental values of 3.843, 4.024, 4.00 (the bulk lattice parameter of $SrTaO_3$ is not well known), and 3.900 Å, respectively. For all interface calculations, a 1×1×14 perovskite unit cell superlattice was used. With a stoichiometry equivalent to 7 layers of $SrTiO_3$ and 7 layers of $SrBO_3$ (where B=V, Nb, and Ta). The in-plane lattice constants a and b were constrained to the theoretical value of $SrTiO_3$ (3.879 Å), while the out-of-plane lattice vector c was optimized within the P4mm space group. Ionic coordinates were optimized until all Hellman-Feynman forces were less than 5 meV/Å. Reported conduction electrons were computed from states from E_F to the gap defined by the nominally O2*p* states.

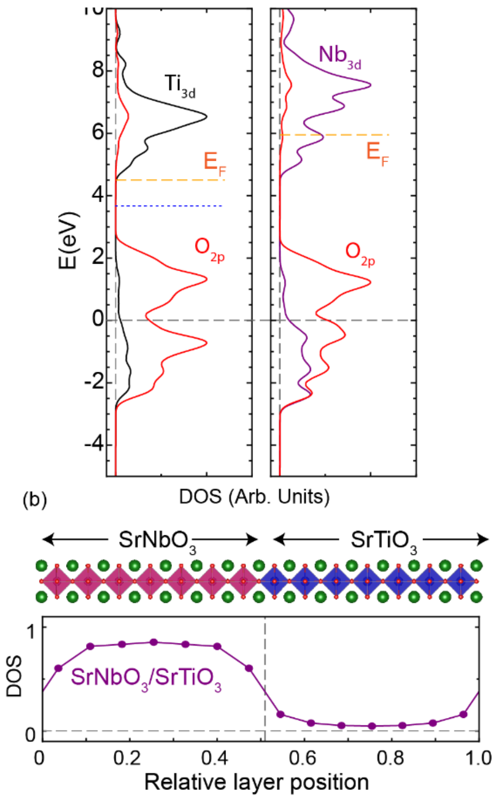

Figure S2. (a) Energy dependent density of states (DOS) for bulk $SrTiO_3$ (left), $SrNbO_3$ (right) where the energy has been shifted to align the O2*p* states at *E* = 0 eV which is denoted by a dashed gray line. The red curves are the O2*p* states, and the black/purple curves are the transition metal *d*-states, as indicated. The orange line indicates the Fermi energy (the dotted blue line for $SrTiO_3$ indicates the Fermi energy in the intrinsic limit). (b) Spatially resolved layer-by-layer # electrons for superlattice systems. Data points indicate Nb-site positions within a supercell as indicated by the crystal structure model vertically above. Left of the dashed vertical line is the $SrNbO_3$ side of the interface, and the right side is the $SrTiO_3$. The non-zero number of electrons on the $SrTiO_3$ indicates the presents of a 2DEG due to charge transfer into the $SrTiO_3$ from $SrNbO_3$.

Multicarrier transport model

A multicarrier model for the Hall effect can be used to gain insight into the origin of the observed non-linearity in the $SrTaO_3/SrTiO_3$ system. The mathematical functions describing the Hall resistance and magnetoresistance of a multiband metal as a function of the areal carrier densities (n_i) and mobilities (μ_i) can be derived by considering parallel channels. As such, the conductance is simply the summation of the individual components:

$$G_{xx} = \sum_i \frac{e|n_i|\mu_i}{1+\mu_i^2 B^2}$$

$$G_{xy} = \sum_i \frac{e n_i \mu_i^2 B}{1+\mu_i^2 B^2}$$

where e is the electron charge and B is the magnetic field. Then the sheet and the Hall resistivities are given, respectively, by

$$R_{xx} = \frac{G_{xx}}{G_{xx}^2 + G_{xy}^2},$$

and

$$R_{xy} = \frac{G_{xy}}{G_{xx}^2 + G_{xy}^2}.$$

The conductivities and resistivities are related to the conductance and resistances as $\sigma = G/thickness$ and $\rho = R*thickness$.

This provides a set of equations that can be used to fit experimental Hall effect data, with 2 parameters (n and μ) per conductance channel. For the case of $SrTaO_3$ on $SrTiO_3$ the resistivity versus temperature analysis in the main text shows that the $SrTiO_3$ substrate contributes to the transport with a carrier density in the range of 10^{16}-10^{17} cm^{-3}, which implies that there are at least two conductance channels. A third channel emerges due to populating the interfacial $SrTiO_3$, which is consistent with the band alignment prediction as well as experimentally consistent with the nonlinearity observed in the Hall effect. This 3-channel model requires 6 independent parameters. However, the well-established transport phenomena of $SrTiO_3$, and the independent measurements of $SrTaO_3$ on highly-insulating $GdScO_3$ enables us to confine to 3 parameters, as follows. Based on the analysis of resistivity versus temperature in the main text shows that $SrTiO_3$ in the estimated carrier density range has a mobility of the order of 1,000 $cm^2V^{-1}s^{-1}$, which we take to be roughly this value. $SrTaO_3$ has a carrier density roughly 1.7×10^{22} cm^{-3} (1 electron per unit cell), and a mobility of order 1 $cm^2V^{-1}s^{-1}$. As such, we let the carrier density of the $SrTiO_3$ be a fit parameter as well as the carrier density and mobility of the 2DEG state that is responsible for the hole-like behavior. This reduces 6 parameters to 3 parameters and enables us to better understand the unusual behavior observed for the $SrTaO_3/SrTiO_3$ system. We stress that due to the complex band structure and scattering processes the multicarrier analysis only gives a semi-qualitative understanding of the different transport channels, and the estimated error in the stated numbers is likely large. As such, we fit the experimental data only when there is a strong non-linearity (<30 K for the 7.6 nm $SrTaO_3/SrTiO_3$ film) and estimate how the various transport parameters change which describes the higher temperature regime and thicker films.

First, as discussed in the main text and shown in Figure 4, a strong non-linear Hall effect is found for thin samples at low temperature. For thicker samples and higher temperatures, the non-linearity is

reduced. For the 7.6 nm sample the non-linearity is absent at 300 K, and with reducing temperature is peaked around 8 K, and with further reducing temperature the magnitude steadily drops down to 2 K. As such, we first fit the 2 K data, with the results shown in Figure S3 and the parameters in Table S1. The fit agrees excellently with the experimental data, which yields an $SrTiO_3$ carrier density in the range of 10^{16} cm^{-3} and an areal carrier density of the 2DEG state of order of 10^{13} cm^{-2} (to extract the volume carrier density a thickness of 5 nm was assumed) and a 2DEG mobility of order 3700 $cm^2V^{-1}s^{-1}$. These values are in line with typical values measured for $SrTiO_3$ as well as 2DEGs, and further represents a reasonable amount of charge that may be transferred across an interface.

Secondly, to understand why the overall slope drops for large film thicknesses we note the following. First, the areal carrier density of the $SrTaO_3$ increases, but also the carrier density of the $SrTiO_3$ substrate increases as well. This is due to more oxygen vacancies being produced due to the longer dwell time at the growth temperature required. Hence, in Figure S3(a), we simulated this by increasing both the areal density of $SrTaO_3$ as well as $SrTiO_3$. What can be seen is the overall Hall slope drops with increasing $SrTiO_3$ carrier density. This exactly reproduces the experimental trend.

Thirdly, at higher temperatures the slope of R_{xy} for the 7.6 nm film drops. This is most likely driven by a change to the mobilities in bulk $SrTiO_3$ and 2DEG systems, where the mobilities are highly temperature dependent. As such, in Figure S3(b) we simply lowered the mobility of both the $SrTiO_3$ and the 2DEG state by a factor of 7, where the mobilities are of order of 100. At this value the simulations shows that this reduces the slope of R_{xy} and eliminates the non-linearity, which exactly agrees with the room-temperature experimental data shown in this figure. We note that these mobilities are higher than typical values at room temperature (typically of order of 10 $cm^2V^{-1}s^{-1}$), and, in fact, as noted below, this may be confounded with a change in carrier density of $SrTiO_3$ at elevated temperatures since the Fermi energy is smaller than the temperature scale). This again highlights the semi-qualitative nature of our model and stresses that these changes should only be interpretated as order-of-magnitude estimations.

Finally, the non-monotonic change of the Hall slope with reducing temperature for the 7.6 nm $SrTaO_3/SrTiO_3$ is very unusual. To see this, compare the data shown in Figure S3(a) at 2 K to the data in Figure S3(c) which was measured at 8 K. To estimate the change in parameters, we performed the same fitting procedure, which, as shown in Table S1, shows an increase in the carrier density of the 2DEG (1.5×10^{13} $cm^{-2}\rightarrow2.2\times10^{13}$ cm^{-2}), a slight reduction in the 2DEG mobility (3700 $cm^2V^{-1}s^{-1}\rightarrow$3300 $cm^2V^{-1}s^{-1}$), and the $SrTiO_3$ carrier density is nearly constant. A second fit is shown where the 2DEG values were fixed at those of the 2 K fit, and only the $SrTiO_3$ parameters were varied. The rational is that at a very dilute density these parameters are known to both be dependent on temperature (see for example Figure 3 in Ref. [5]), which can be rationalized since the Fermi energy is of order the temperature (i.e. $E_F = \hbar^2(3\pi^2 n)^{2/3}/(2m^*) \approx 2$ K, where $\hbar$ is Planck's reduced constant, and m^* is the effective mass, which is well known to be of order the electron mass for $SrTiO_3$). Here the fit is equally good and shows that the carrier density and mobility slightly decrease in going from 2 K to 8 K, which is consistent with the magnitude of changes observed in bulk $SrTiO_3$. However, the order-of-magnitude of the fit parameters are likely correct, yet the finer changes are likely within the error bars (since two models with different parameters yield excellent fits); therefore, it is likely not meaningful to scrutinize the physical origins of the relative changes.

Overall, the multicarrier analysis shows that for the $SrTaO_3/SrTiO_3$ that the interface must be populated by charge transfer from the $SrTaO_3$. More specifically, the transport can only be explained by the high carrier density $SrTaO_3$ and two high mobility states, which can only be the lightly doped $SrTiO_3$ substrate and an interfacial 2DEG. In contrast, the $SrVO_3/SrTiO_3$ system clearly shows no charge transfer,

and the transport is solely through the $SrVO_3$. This together is fully supported by the band alignment analysis, which predicts that $SrTaO_3$ forms an Ohmic-type interface with $SrTiO_3$ and $SrVO_3$ forms a Schottky-type interface.

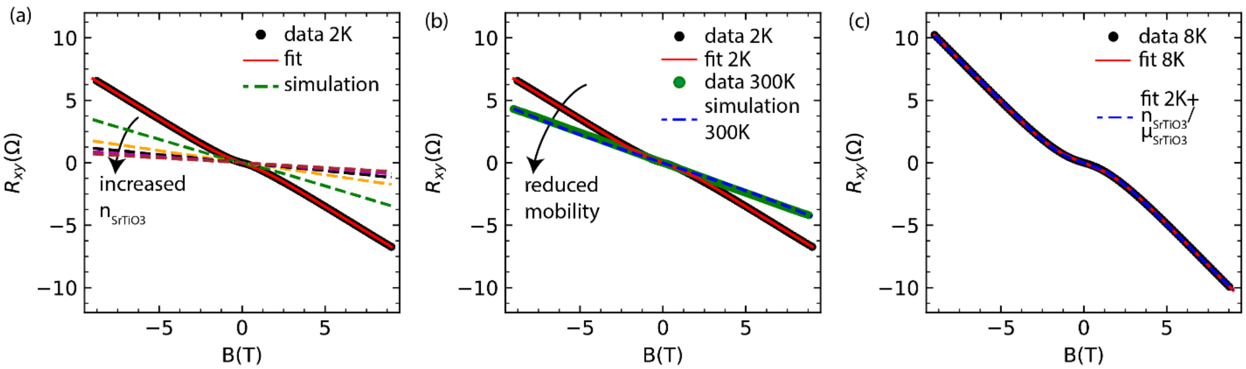

Figure S3. Data, multicarrier fits, and simulations for the 7.6 nm $SrTaO_3$ for various temperatures. (a) Data taken at 2 K, fit, and simulations with increasing the $SrTiO_3$ carrier density which reproduces the trend for thicker $SrTaO_3$/$SrTiO_3$ as shown in Figure 4 of the main text. (b) Data taken at 2 K and fit, as well as data taken at 300 K. Simulation corresponding to the parameters of the 2 K fit with mobility reduced, which reproduces the 300 K data. (c) Data taken at 8 K, which shows a larger R_{xy} relative to 2 K, which is fit as in (a), red, as well as by taking the 2DEG fit parameters from 2 K then varying the $SrTiO_3$ carrier density and mobility.

		$SrTiO_3$		$SrTaO_3$		2DEG		
Panel/color	Type	n(cm^{-3})	μ ($cm^2V^{-1}s^{-1}$)	n(cm^{-3})	μ ($cm^2V^{-1}s^{-1}$)	n(cm^{-3})	n(cm^{-2})	μ ($cm^{-2}V^{-1}s^{-1}$)
(a)/red	fit	-8.0E+15	1000	-1.7E+22	1	3.0E+19	1.5E+13	3700
(a)/green	simulation	-1.6E+16	1000	-1.7E+22	1	3.0E+19	1.5E+13	3700
(a)/orange	simulation	-3.2E+16	1000	-1.7E+22	1	3.0E+19	1.5E+13	3700
(a)/black	simulation	-4.8E+16	1000	-1.7E+22	1	3.0E+19	1.5E+13	3700
(a)/magenta	simulation	-6.4E+16	1000	-1.7E+22	1	3.0E+19	1.5E+13	3700
(a)/brown	simulation	-8.0E+16	1000	-1.7E+22	1	3.0E+19	1.5E+13	3700
(b)/red	fit	-8.0E+15	1000	-1.7E+22	1	3.0E+19	1.5E+13	3700
(b)/blue	simulation	-1.0E+16	140	-1.7E+22	1	4.1E+20	2.0E+14	500
(c)/red	fit	-5.0E+15	1000	-1.7E+22	1	4.3E+19	2.2E+13	3300
(c)/blue	fit	-5.0E+15	860	-1.7E+22	1	3.0E+19	1.5E+13	3700

Table S1. Parameters of the multicarrier fit and simulations shown in Figure S3. The carrier density for the channel associated with a 2DEG is shown in 3D density (assuming ~5 nm thickness).

Additional transport data

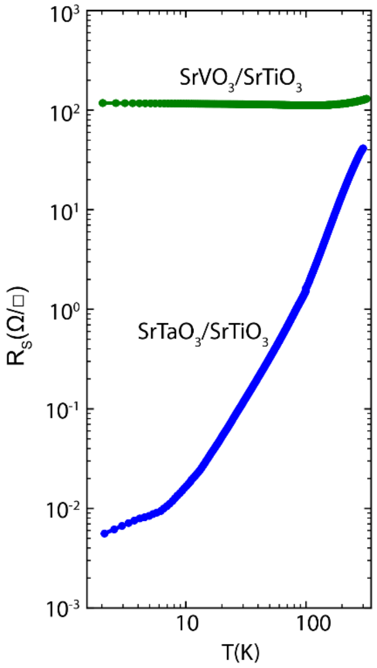

Figure S4. Temperature dependence of longitudinal sheet resistance for $SrVO_3/SrTiO_3$ (16 nm) and $SrTaO_3/SrTiO_3$ (14 nm).

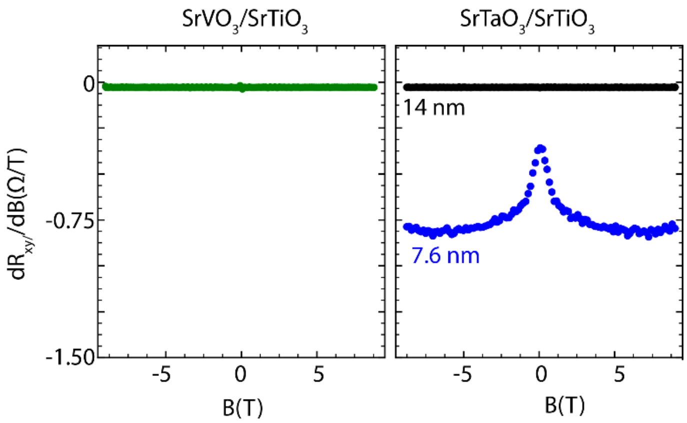

Figure S5. Derivative for the Hall effect (dR_{xy}/dB), where $SrVO_3$ is constant (c) whereas $SrTaO_3/SrTiO_3$ shows non-linear behavior. This data corresponds to the same samples as show in Figure 4 of the main text.

References

[1] P. Giannozzi, S. Baroni, N. Bonini, M. Calandra, R. Car, C. Cavazzoni, D. Ceresoli, G. L. Chiarotti, M. Cococcioni, I. Dabo, A. D. Corso, S. de Gironcoli, S. Fabris, G. Fratesi, R. Gebauer, U. Gerstmann, C. Gougoussis, A. Kokalj, M. Lazzeri, L. Martin-Samos, N. Marzari, F. Mauri, R. Mazzarello, S. Paolini, A. Pasquarello, L. Paulatto, C. Sbraccia, S. Scandolo, G. Sclauzero, A. P. Seitsonen, A. Smogunov, P. Umari, and R. M. Wentzcovitch, J. Phys. Condens. Matter **21**, 395502 (2009).

[2] S. Okamoto and A. J. Millis, Nature **428**, 630 (2004).

[3] V. R. Cooper, S. S. A. Seo, S. Lee, J. S. Kim, W. S. Choi, S. Okamoto, and H. N. Lee, Sci. Rep. **4**, 1 (2014).

[4] V. R. Cooper, Phys. Rev. B **85**, 235109 (2012).

[5] A. Spinelli, M. A. Torija, C. Liu, C. Jan, and C. Leighton, Phys. Rev. B **81**, 155110 (2010).
